# Comparing causes of death between formal and informal neighborhoods in urban Africa: evidence from Ouagadougou Health and Demographic Surveillance System

**DOI:** 10.3402/gha.v7.25523

**Published:** 2014-10-29

**Authors:** Abdramane Bassiahi Soura, Bruno Lankoande, Roch Millogo, Martin Bangha

**Affiliations:** 1 Ouagadougou Health and Demographic Surveillance System, Institut Supérieur des Sciences de la Population, Université de Ouagadougou, Ouagadougou, Burkina Faso; 2 INDEPTH Secretariat, Accra, Ghana

**Keywords:** causes of death, verbal autopsy, Ouagadougou, formal and informal neighborhoods

## Abstract

**Background:**

The probable coexistence of two or more epidemiological profiles in urban Africa is poorly documented. In particular, very few studies have focused on the comparison of cause-specific mortality between two types of neighborhoods that characterize contemporary southern cities: formal neighborhoods, that is, structured or delineated settlements (planned estates) that have full access to public utilities (electricity and water services), and the informal neighborhoods, that is, spontaneous and unplanned peri-urban settlements where people live in slum-like conditions, often with little or no access to public utilities.

**Objective:**

To compare the causes of death between the formal and informal neighborhoods covered by the Ouagadougou Health and Demographic Surveillance Systems (HDSS).

**Design:**

The data used come from the INDEPTH pooled dataset which includes the contribution of Ouagadougou HDSS and are compiled for the INDEPTH Network Data repository. The data were collected between 2009 and 2011 using verbal autopsy (VA) questionnaires completed by four fieldworkers well trained in the conduction of VAs. The VA data were then interpreted using the InterVA-4 program (version 4.02) to arrive at the causes of death.

**Results:**

Communicable diseases are the leading cause of death among children (aged between 29 days and 14 years) in both formal and informal neighborhoods, contributing more than 75% to the mortality rate. Mortality rates from non-communicable diseases (NCDs) are very low before age 15 but are the leading causes from age 50, especially in formal neighborhoods. Mortality from injuries is very low, with no significant difference between the two neighborhoods.

**Conclusions:**

The fact that mortality from NCDs is higher among adults in formal neighborhoods seems consistent with the idea of a correlation between modern life and epidemiological transition. However, NCDs do affect informal neighborhoods as well. They consist mainly of cardiovascular diseases and neoplasms most of which are preventable and/or manageable through a change in lifestyle. A prevention program would certainly reduce the burden of these chronic diseases among adults and the elderly with a significant economic impact for families.

Demographic transition, defined as the decline in mortality and fertility resulting from socio-economic development, is usually followed by epidemiological transition. The latter is the evolution in which infectious and parasitic diseases, as leading causes of morbidity and death, are gradually replaced by chronic conditions. All industrialized countries have gone through this process that began since the 1800s (1). The developing countries are currently at varying stages in this epidemiological transition (2). In some regions, such as sub-Saharan Africa, the stages overlap, since the resurgence of some infectious diseases (meningitis, malaria …) and HIV/AIDS co-exist with the increase of chronic diseases (2). Sub-Saharan Africa is therefore facing a ‘double burden’, especially at adult ages (2, 3).

Determinants of epidemiological transition are intimately associated with those of demographic transition and are generally attributed to ‘modernization’. In developing countries, the epidemiological transition is correlated with the urban transition. Indeed, urbanization, depending on its speed and intensity, may result in environmental change, which in turn can influence behaviors such as physical activity (4). For instance, the mechanization of certain tasks (at home and at work) and the availability of intra-urban means of travelling most often results in some amount of physical inactivity. Furthermore, some work-related physical activities or tasks may lose their value because of e-administration. Moreover, urban transition is usually followed by nutrition transition, which is the transition from traditional (natural) nutritive diets toward western-style diets (particularly fast foods), rich in processed products. Indeed, with industrialization of food, globalization of trade, advertising and marketing of agro-food industries, urban dwellers are increasingly exposed to massive imports of manufactured food products (5). The nutrition transition in turn leads to a health transition marked by the emergence of overweight and obesity, hypertension, diabetes, hypercholesterolemia, and increased mortality from cardiovascular diseases and some cancers (5).

Several studies in developing countries have shown a higher risk of chronic diseases among urban adults compared to their rural counterparts (6–12). Comparative studies on causes of death are rare due to the absence of population-specific surveys on the topic. In most cases, data on causes of death are from hospital statistics, which are severely affected by selection bias given that most deaths in developing countries occur outside the hospital system and clinical autopsies are almost never performed. One of the few leading works in the field is the study by Walker et al. that compares stroke mortality in rural and urban Tanzania using data from three demographic surveillance systems (13). They found that stroke mortality, especially among urban women, was significantly higher than in rural areas because of the advanced epidemiological transition in the city (13).

In Burkina Faso, as in most sub-Saharan African countries, evidence on the epidemiological transition can be pieced together only for selected parts of the country where Health and Demographic Surveillance Systems (HDSS) exist. Four of the five currently operational HDSSs in the country are located in areas that are predominantly rural. Studies on cause-specific mortality in urban areas, and more importantly within the same city, are thus virtually not found in Burkina Faso as in most developing countries. The probable coexistence of two or more epidemiological profiles in urban areas is thus poorly documented. This paper is a contribution to knowledge in this area by taking advantage of data from the Ouagadougou HDSS to compare the causes of death between two types of neighborhoods that characterize contemporary southern cities: formal neighborhoods and informal neighborhoods (or settlements). Formal neighborhoods are structured or delineated settlements (planned estates), with full access to public utilities (electricity and water services), while informal neighborhoods are mainly spontaneous settlements that sprang up in these cities as a result of rapid and ‘uncontrolled’ urbanization. Their inhabitants often live in abject poverty with no means to secure adequate housing in formal neighborhoods.

This paper on cause-specific mortality is mainly descriptive work in which we expect more non-communicable diseases (NCDs), that is, diseases that cannot be passed from person to person as adult causes of death in formal neighborhoods compared to the informal ones. In the latter, we expect a mixed epidemiological profile for adults; NCDs and communicable diseases (CDs), that is, transmitted through direct contact with an infected individual or indirectly through a vector, should be of similar magnitude. Indeed, social and dietary changes that accompany urbanization and expose people to NCDs are much more noticeable in formal neighborhoods. In informal neighborhoods, poor living conditions place the population at a high risk of CDs but at the same time, people, because of their poverty, may quickly adopt ‘deviant’ behaviors such as excessive drinking and smoking, two NCD risk factors (14). Urban poor may also consume more energetically dense foods because they seem inexpensive but which, combined with physical inactivity, expose them more to NCDs (15).

In the case of children, one will normally not expect any notable difference in the causes of death according to the type of neighborhood. Although all age groups can be affected by NCDs, these diseases are often associated with older age groups. In developing countries, children die more from CDs whatever the place of residence (16). Even urban Ouagadougou is not different in this regard and so the expectation in this study is for mortality in children to be dominated by CDs regardless of the type of neighborhood.

## Data and methods

This work contributes to enrich the scientific debate on intra-urban differences in the epidemiological profile through an analysis of causes of death. The latter are compared between the formal and informal neighborhoods covered by the Ouagadougou HDSS.

### Study population

Data used come from the INDEPTH pooled dataset which includes the contribution of Ouagadougou HDSS and compiled for the INDEPTH Network Data repository (17). The Ouagadougou HDSS is a platform for health research and interventions established in 2008 covering five neighborhoods of Ouagadougou (18). Two of these neighborhoods (Kilwin and Tanghin) are formal neighborhoods with full access to public services, while the other three (Nonghin, Polesgo, and Nioko 2) are spontaneous (such as slums) without access to such services. People living in informal areas are poorer on average, less educated, and born in rural areas in comparison with people living in formal settlements (19), which highlights the importance of rural outmigration to the growth of informal urban settlements. Households in the informal settlements are usually small, made up of single men or young nuclear families in search of affordable housing (19).

After an initial census conducted between October 2008 and March 2009 in the five neighborhoods, fieldworkers make regular household visits for update rounds (with an average periodicity of 7 months), registering vital events (births and deaths, marriages, and migrations). As at November 2012, the population under surveillance by the Ouagadougou HDSS totaled 86,071 residents (defined as individuals present in the zone for at least 6 months). In case of death, a verbal autopsy (VA) questionnaire is completed with the next of kin to determine the circumstances that led to the death, including history of the illness and the specific symptoms that preceded death. It should be noted that although the data used come from the INDEPTH pooled dataset, not all INDEPTH members used the INDEPTH standard VA instrument. In 2012, a group of experts under the auspices of WHO reviewed the existing VA instruments in the world and proceeded to their simplification and their standardization to make the results comparable (20).

A revised list of causes of death has been established by grouping all ICD-10 causes of death into 62 broad categories. These categories were chosen on the basis of their public health relevance and their potential for ascertainment from VA. A total of 245 indicators (questions) were included in the revised VA instrument. A matrix of these indicators is the input file for the InterVA-4 model used for processing VA data to produce CoD for analysis in this special issue; all the contributing HDSSs transformed their CoD data into this matrix for use in the version 4.02 of InterVA-4 (21). This model applies Bayesian probabilistic methods to VA data and arrives at possible causes of death (21). It generates a maximum of three likely causes of death per case with their associated partial likelihoods (between 0 and 1). For some cases, the input data are insufficient for InterVA-4 to generate any cause of death and such cases are classified by InterVA-4 into the ‘indeterminate’ cause of death. For each case where the sum of the partial likelihoods does not total 1, the difference between their sum and 1 is assigned to the ‘indeterminate’ cause. For this paper, all identified causes of death will be considered proportionate to their partial likelihoods in the calculation of the number of deaths from each cause.

In this INDEPTH pooled dataset, data from Ouagadougou HDSS cover the period 2009–2011 and include 1,032 deaths recorded across 221,178 person-years. Of the 1,032 recorded deaths, 870 VAs were completed. These VA data are used to compare formal and informal neighborhoods in terms of causes of death. In the corresponding multisite papers presented in this special issue, the Ouagadougou results are presented as one site.

### Indicators and methods

This study examined mortality rates, proportion of deaths due to each cause, and the contribution of each cause to the all-cause mortality rate. Mortality rates are obtained by dividing the number of deaths by the number of person-years. Our estimates will not provide confidence intervals since the HDSS covers an entire non-sampled population. Due to small number of deaths involved, the mortality rates are calculated only for major groups of causes (CDs, NCDs, maternal and neonatal causes, injuries, and unspecified causes). These groups are predefined in the InterVA-4 model (version 4.02) used. CDs include diarrheal diseases, HIV/AIDS, non-obstetric sepsis, malaria, meningitis and encephalitis, respiratory infections, TB, and other infectious diseases. The most common NCDs are anemia, asthma, cardiovascular diseases, neoplasms, diabetes, renal failure, acute abdomen, epilepsy, and severe malnutrition. Maternal and neonatal mortality includes by implication pregnancy-/birth-related causes (pregnancy-induced hypertension, pregnancy-related sepsis, obstetric hemorrhage) and neonatal causes (prematurity, birth asphyxia, neonatal pneumonia, neonatal sepsis, and congenital malformation).

To better portray the cause-specific mortality by age, we used the seven age groups predefined in InterVA-4 model (version 4.02), which correspond theoretically to different leading causes of death. Thus, children were grouped into four categories with different levels of exposure to various diseases: neonates (less than 28 days), infants (29 days–11 months), children between 1 and 5, and those between 5 and 15. Among adults, the elderly (65 and over) have been distinguished from people aged 50–64 and from those aged 15–49.

Table 1 presents the person-years distribution by age group, sex, and neighborhood, although the small number of deaths here does not allow us to perform mortality analysis by sex. For each sex, formal and informal neighborhoods have close distributions. Regardless of gender and type of neighborhood, people aged 15–49 are the most represented, accounting for more than 50%, followed by those aged 5–14 representing just over 1 in 5. The proportion of people aged 15–49 is slightly higher in formal neighborhoods. There are relatively more children (1–11 months and 1–4 years) in informal neighborhoods while older people (50 years and older) are slightly more in formal neighborhoods. To control for this slight difference in age structure between formal and informal neighborhoods, we provide standardized mortality rates next to the crude mortality rate (all ages) for each type of neighborhood. For this purpose, we have used the structure of the two types of neighborhoods combined as the standard population.

**Table 1:** Distribution (%) of person-years by age group, sex, and neighborhood, 2009–2011

	Formal neighborhood		Informal neighborhood		Total	
			
Age group	Male	Female	Male	Female	Male	Female
0–28 days	0.2%	0.1%	0.2%	0.3%	0.2%	0.2%
29 days–11 months	2.1%	2.1%	3.7%	3.9%	2.9%	3.0%
1–4 years	10.0%	9.8%	15.7%	15.6%	12.7%	12.5%
5–14 years	22.4%	23.7%	22.2%	24.4%	22.3%	24.0%
15–49 years	56.0%	56.0%	53.2%	50.5%	54.6%	53.4%
50–64 years	7.0%	5.9%	4.0%	3.6%	5.6%	4.8%
65+ years	2.3%	2.4%	1.0%	1.7%	1.7%	2.1%
Person-years	57500.5	57932.8	53492.4	52252.4	110992.9	110185.2

## Results

### All-age mortality

In the study area, the overall crude mortality rate is estimated at 4.7 per 1,000 person-years. It is 4.2 per 1,000 person-years in the formal neighborhoods and 5.2 per 1,000 person-years in the informal ones. Despite the standardization to account for the difference in age structure, the excess mortality remains in informal neighborhoods with a mortality rate of 4.6 per 1,000 persons-years against 3.9 for formal neighborhoods. All ages combined, CDs are the leading causes of death in the Ouagadougou HDSS. Indeed, the mortality rate from CDs is estimated at 1.71 per 1,000 person-years against 1.22 for NCDs, 0.19 for injuries (see Table 3). Mortality from CDs is more prevalent in informal neighborhoods with an age-adjusted rate of 2.11 per 1,000 person-years against 1.22 in the formal ones. In the latter, the risk of death associated with NCDs is close to that related to CDs (1.32 per 1,000 person-years against 1.22 per 1,000 person-years as age-adjusted rates). There is little difference between the two types of neighborhoods in terms of injuries-related mortality risk (age-adjusted rate of 0.22 per 1,000 person-years for informal neighborhoods against 0.16 per 1,000 person-years for the formal ones).

**Fig. 1:**
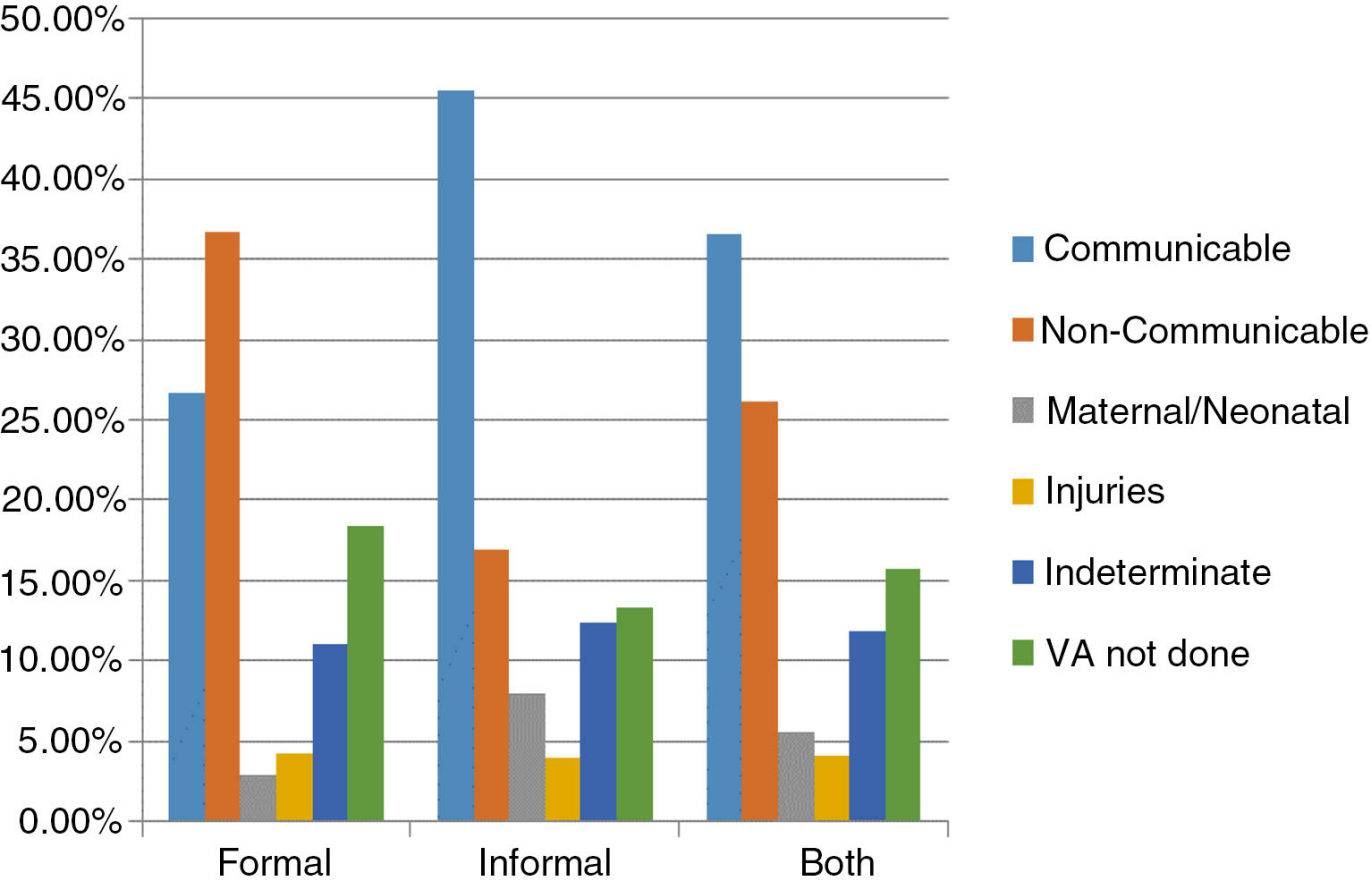
Percentage of people dying from each group of causes according to the type of neighborhood (Percentage calculated on 485 deaths in formal neighborhoods and 547 deaths in informal neighborhoods).

In terms of proportion (Fig. 1), CDs are responsible for about 27% of all deaths that occurred in formal neighborhoods and NCDs responsible for 37% in the same neighborhoods. The situation is reversed in informal neighborhoods where CDs account for more than 45% of all deaths against about 17% for NCDs.

Among the CDs, malaria is the top cause of death as in many sub-Saharan African countries. It was responsible for about 11.7% of all-ages deaths in the study site, 7.1% in formal neighborhoods and 15.8% in informal neighborhoods (Table 2). Second to malaria, in order of importance are acute respiratory infections, HIV/AIDS, and diarrheal diseases with 10.0, 5.8 and 3.2% of deaths, respectively. NCDs consist mainly of cardiovascular diseases which accounted for 17.7% of deaths in formal neighborhoods and 5.7% in informal neighborhoods. Mortality from neoplasms, taken separately, is also more prevalent in formal neighborhoods (11.9% of deaths) than in the informal ones (4.7% of deaths) while mortality from asthma, diabetes, and anemia is low, accounting for less than 1%.

**Table 2:** Cause-specific mortality fraction (%) by neighborhood, 2009–2011

Cause of death	Formal (*n*=485) (%)	Informal (*n*=547) (%)	Both (*n*=1,032) (%)
**Communicable diseases (CDs)**	**26.6**	**45.5**	**36.6**
Sepsis (non-obstetric)	0.8	0.4	0.6
Acute resp infect incl. pneumonia	8.0	11.7	10.0
HIV-/AIDS-related death	4.8	6.7	5.8
Diarrheal diseases	1.2	4.9	3.2
Malaria	7.1	15.8	11.7
Meningitis and encephalitis	2.1	3.1	2.6
Pulmonary tuberculosis	2.4	2.1	2.2
Other and unspecified infect dis	0.2	0.8	0.5
**Non-communicable diseases (NCDs)**	**36.7**	**16.9**	**26.2**
Neoplasms	11.9	4.7	8.1
Severe anemia	0.3	0.3	0.3
Severe malnutrition	1.8	3.1	2.5
Diabetes	0.8	0.2	0.5
Cardiovascular diseases	17.7	5.7	11.3
Asthma	0.3	0.2	0.2
Acute abdomen	3.1	2.3	2.7
Renal failure	0.0	0.1	0.0
Epilepsy	0.4	0.3	0.4
Other and unspecified NCDs	0.4	0.0	0.2
**Maternal/neonatal**	**2.9**	**7.9**	**5.6**
Maternal causes	0.8	0.4	0.6
Neonatal causes	2.1	7.5	5.0
**Injuries**	**4.3**	**3.9**	**4.1**
**Indeterminate**	**11.1**	**12.5**	**11.8**
**VA not done**	**18.4**	**13.3**	**15.7**

### Age-specific mortality

The risk of dying from CDs for children is between 1 and 5 times higher in informal neighborhoods than in the formal ones (Table 3). The child mortality rate from this cause is estimated at 6.18 per 1,000 person-years in the informal neighborhoods against 3.09 per 1,000 person-years in the formal ones. For infants aged between 29 days and 11 months, an excess mortality from CDs is clearly visible in informal neighborhoods with a rate of 17.08 per 1,000 person-years against 9.17 per 1,000 person-years in formal neighborhoods. For older age groups (children aged 5–14, adults aged 15–49, those aged 50–64, or the elderly, i.e., aged 65+), mortality rates due to CDs in formal and informal neighborhoods are close though relatively higher in informal neighborhoods (Table 3).

**Table 3:** Cause-specific mortality rates (per 1,000 person-years) by neighborhood and age group, 2009–2011

	CDs			NCDs		
		
	Both	Formal	Informal	Both	Formal	Informal
Neonatal^a^	6.92	4.07	8.54	0.00	0.00	0.00
29 days–11 months	14.12	9.17	17.08	1.29	0.31	1.88
1–4 years	4.92	3.09	6.18	0.93	0.44	1.26
5–14 years	0.70	0.51	0.91	0.11	0.08	0.13
15–49 years	0.47	0.44	0.50	0.63	0.68	0.57
50–64 years	2.63	2.06	3.69	5.87	7.50	2.84
65+ years	5.79	5.02	7.27	21.29	25.76	12.70
**All ages crude rate**	**1.71**	**1.12**	**2.35**	**1.22**	**1.54**	**0.87**
**Age-adjusted rate**		**1.22**	**2.11**		**1.32**	**0.94**
	Maternal/neonatal			Injuries		
		
	Both	Formal	Informal	Both	Formal	Informal

Neonatal^a^	91.18	55.37	111.61	0.00	0.00	0.00
29 days–11 months	0.64	0.00	1.02	0.15	0.00	0.24
1–4 years	0.03	0.00	0.05	0.11	0.09	0.12
5–14 years	0.01	0.01	0.00	0.13	0.04	0.22
15–49 years	0.05	0.06	0.04	0.14	0.13	0.15
50–64 years	0.00	0.00	0.00	0.57	0.67	0.40
65+ years	0.00	0.00	0.00	1.99	1.94	2.10
**All ages crude rate**	**na**	**na**	**na**	**0.19**	**0.18**	**0.20**
**Age-adjusted rate**		**na**	**na**		**0.16**	**0.22**

na=non-applicable. Not calculated because all age groups or all sexes are not exposed to the risk of maternal mortality. At the same time, all age groups are not exposed to a risk of death from neonatal causes.

a Rates in the Table are calculated per 1,000 person-years and hence may appear very high for neonatal mortality since each neonate cannot contribute more than one-twelfth of a person-year each.

Mortality rates from NCDs are relatively low before age 50, which is logical given that these diseases are strongly associated with age. Beyond this age, the difference between formal and informal neighborhoods is large. The mortality rate is at least two times higher in formal neighborhoods (Table 3). For instance, it is 25.76 per 1,000 person-years among people aged 65 and over against 12.70 per 1,000 person-years for their counterparts in informal neighborhoods. Deaths from injuries are very rare and do not suggest any significant difference between the two types of neighborhoods (Table 3). Neonatal causes are two times higher in informal neighborhoods compared to the formal ones (Table 3).

**Fig. 2:**
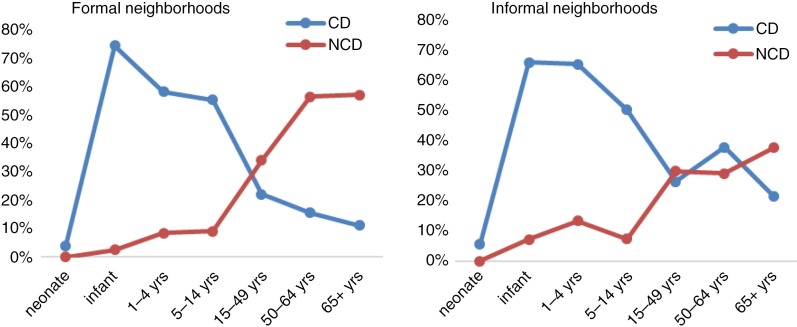
Mortality fraction from communicable diseases or non-communicable diseases by age group and type of neighborhood.

Figure 2 provides a better illustration of the contrast between CDs and NCDs in terms of leading cause of death by age group and neighborhood. As can be observed, before age 15, the proportion of deaths attributable to CDs is higher than that of NCD-related deaths whatever the type of neighborhood. The only exception is for the neonates where the two groups of causes have quite similar proportions. Among adults (aged 15 and over), two distinct situations are noticeable depending on the type of neighborhood. In formal neighborhoods, the proportion of deaths due to NCDs is far higher than that of deaths from CDs while in informal neighborhoods, the situation is mixed. Indeed, for informal neighborhoods, the proportion of NCDs and CDs are close before the age 65, but from 65 years, NCDs become more pronounced compared to CDs.

This analysis of mortality fractions was complemented by calculating the contribution of each group of causes (among known causes) to the total risk of death (Table 4). The results confirm that CDs are the leading causes of death among children aged 29 days to 14 years, regardless of the type of neighborhood. For instance, among the known causes, CDs are responsible for 87.2% of the risk of death for infant aged 29 days–11 months. This proportion is 84.5% in informal neighborhoods and 96.7% in the formal ones. Among children 1–4 years, this proportion is estimated at 82.2% and does not vary much according to the type of neighborhood; for those aged 5–14, it is 79.4% in formal neighborhoods and 71.8% in the informal ones.

**Table 4:** Contributions (%) of the grouped causes to the risk of death by neighborhood and age group, 2009–2011

Group of causes	Type of neighborhood	Neonatal	29 days–11 months	1–4 years	5–14 years	15–49 years	50–64 years	65+ years	Under 15 years	15+ years	All ages
	Both	7.0	87.2	82.2	74.5	36.4	29.1	19.9	72.4	29.1	50.5
Communicable	Formal	6.8	96.7	85.3	79.4	33.7	20.2	15.4	77.6	23.0	37.7
diseases	Informal	7.1	84.5	81.1	71.8	39.8	53.2	32.9	70.6	41.0	61.3
	Both	0.0	8.0	15.5	11.4	48.8	64.6	73.2	10.7	61.0	36.1
Non-communicable	Formal	0.0	3.3	12.3	12.9	51.8	73.3	78.7	8.7	68.0	52.0
diseases	Informal	0.0	9.3	16.6	10.5	45.0	41.0	57.6	11.4	47.2	22.7
	Both	93.0	3.9	0.5	0.6	3.7	0.0	0.0	14.1	1.5	7.7
Maternal/neonatal	Formal	93.2	0.0	0.0	1.8	4.3	0.0	0.0	11.5	1.5	4.2
	Informal	92.9	5.0	0.7	0.0	2.9	0.0	0.0	14.9	1.6	10.7
	Both	0.0	0.9	1.8	13.5	11.1	6.3	6.9	2.8	8.4	5.7
Injuries	Formal	0.0	0.0	2.4	5.9	10.2	6.5	5.9	2.2	7.5	6.1
	Informal	0.0	1.2	1.6	17.7	12.3	5.8	9.5	3.1	10.2	5.3

Among people aged 15 and over, CDs no longer top the chart as the leading cause of death, all neighborhoods combined (Table 4). Indeed with advancing age, CDs increasingly give way to NCDs, especially in formal neighborhoods. For example, in formal neighborhoods, these diseases account for 73.3% of the mortality risk between 50 and 64 years of age against 20.2% for CDs. NCDs have a contribution of 78.7% to the elderly (65 years and over) mortality rate in formal neighborhoods, against 15.4% for CDs. In informal neighborhoods, none of the two groups of causes contributes more than 50% of adult mortality but the contribution of NCDs remains high (47.2% among people aged 15 and over, Table 4). It is specifically estimated at 45.0% between 15 and 49 years, 41.0% between 50 and 64 years, and 57.6% for people aged 65 and over (Table 4).

## Discussion

In the Ouagadougou HDSS, CDs remain the leading cause of death, accounting for 36.6% of deaths recorded between 2009 and 2011, and contributing 50.5% of the mortality rate. Among these CDs, malaria is the leading cause, followed by acute respiratory infections and HIV/AIDS. For all ages combined, deaths from CDs are more prevalent in informal neighborhoods while deaths from NCDs are more prevalent in formal neighborhoods. Results from the analysis of causes of death by age group are consistent with expectations and confirm our research hypotheses. Indeed, CDs are the leading cause of death among children (aged between 29 days and 14 years) in both formal and informal neighborhoods. There is no apparent difference between formal and informal neighborhoods in the epidemiological profile of children even though childhood mortality is higher in informal neighborhoods. Our results are also consistent with studies in other African urban settings such as Nairobi where Kyobutungi et al. have shown that CDs are the major causes of under-5 mortality in slums, a pattern that may be observable across sub-Saharan Africa (22).

The findings also suggest that among adults (15 years and older), the two types of neighborhoods have different epidemiological profiles. For formal neighborhoods, the profile is dominated by NCDs with a contribution higher than 50% that increases with age. This is very visible among the ages 50–64 and among the elderly (65 and over), with a contribution far greater than that observed for the same age groups in informal neighborhoods. For the latter, the epidemiological profile is mixed, with globally no group of causes accounting for more than 50%. NCDs which consist mainly of cardiovascular diseases and neoplasms exceed the threshold of 50% contribution only for the elderly (65 and over).

The fact that mortality from NCDs is higher in formal neighborhoods confirms the idea of a correlation between urbanization and epidemiological transition. Indeed, the formal neighborhoods of Ouagadougou HDSS are inhabited by wealthier and more educated people. Three adults (15 years and over) out of every four are educated with two in five having at least secondary level (19). These ratios compare to about two in five and one in five in informal neighborhoods (19). To further elucidate with some examples of facilities linked to the standard of living, it is estimated that 54% of households in formal areas have a TV, 17% a refrigerator, 66% a motorcycle, and 10% a car (19). The numbers are respectively 13, 0.8, 30, and 0.6% for informal settlements (19). Moreover, in the formal neighborhoods there are more bars, more shops; in short, this is where one feels the city. A health survey conducted in 2010 in the Ouagadougou HDSS areas showed a considerable prevalence of overweight and hypertension among adults in both neighborhoods, which was significantly higher in formal neighborhoods compared to the informal ones (23).

Prevalence of overweight and hypertension was estimated at 32 and 19.6%, respectively, for formal neighborhoods and at 20 and 13% for the informal ones.

This study is very important for health policy evaluation. Malaria, for instance, is already the subject of a vertical disease control program in Ouagadougou with the distribution of bed nets running for several years. This study shows that malaria remains the leading cause of mortality among CDs. Hence, considerable efforts are still needed to achieve a significant reduction or even eradication of malaria mortality. Meanwhile, NCDs, especially cardiovascular diseases are preventable and/or manageable with a change in lifestyle. A prevention program would probably reduce the burden of these NCDs among adults and the elderly with a significant economic impact for families. In the case of Ouagadougou, such a program should involve both formal and informal neighborhoods as we have seen that both types of neighborhoods are affected by NCDs but with different variants. However, a greater emphasis should be devoted to formal neighborhoods. We believe that a community approach would be effective and useful for the Burkina Faso Health Ministry's new Bureau for NCDs which is currently working on a national communications plan for NCDs. This approach should involve community health workers collaborating with community groups to promote awareness of NCD risk factors and to help individuals make changes in their lives.

This study has some limitations, one of which is the high proportion (15.7%) of deaths without VA. This is partly explained by the migration of family members of the deceased that may be compounded by refusals from others to talk about their deceased relatives. It should also be noted that informal neighborhoods of Ouagadougou are populated mostly by migrants (19) and so may be subject to a selection bias by migration. Indeed, due to their poor living conditions and also because of the low level of social ties in cities (24), many city dwellers of informal neighborhoods may leave the area in the event of chronic conditions in search of care in their villages of origin or in other districts of the city where they may have relations. Other studies have already shown that migrants often tend to return home to die (25, 26). Their eventual death may not be captured by the HDSS. This can partly exaggerate the differences in NCD mortality between formal and informal neighborhoods. A final limitation of the work is the impossibility of extrapolating our findings to the entire city of Ouagadougou as the HDSS only covers five districts including two formal and three informal.

## Conclusions

In sub-Saharan Africa, studies on cause-specific mortality are scarce because of a lack of data on the causes of death. As we have already mentioned, frequently used data from hospital-based statistics on cause-specific mortality are seriously affected by selection bias since most deaths occur outside the formal heath care system with no possibility for clinical autopsy. Only HDSSs that use the VA method can produce ‘reliable’ data on causes of death. Very few among currently existing HDSSs are located in urban areas. The Ouagadougou HDSS is the only one in Africa currently following both formal and informal settlements within a city. These two types of neighborhoods characterize contemporary African cities. The results presented in this paper are an important step toward understanding the intra-urban differences in the epidemiological profile in sub-Saharan Africa. Notwithstanding its limitations, this study provides credible evidence to stimulate discussion on cause-specific mortality differences between formal and informal neighborhoods in urban Africa.
